# Robust radiomics: a review of guidelines for radiomics in medical imaging

**DOI:** 10.3389/fradi.2025.1701110

**Published:** 2026-01-12

**Authors:** Michele Avanzo, Paolo Soda, Marco Bertolini, Andrea Bettinelli, Tiziana Rancati, Joseph Stancanello, Osvaldo Rampado, Giovanni Pirrone, Annalisa Drigo

**Affiliations:** 1 Medical Physics Department, Centro di Riferimento Oncologico di Aviano (CRO) IRCCS, Aviano, Italy; 2 Department of Engineering, University Campus Biomedico of Rome, Rome, Italy; 3 Medical Physics Department, Azienda Unità Sanitaria Locale - IRCCS di Reggio Emilia: Reggio Emilia, Emilia-Romagna, Italy; 4 Medical Physics Department, Veneto Institute of Oncology IOV – IRCCS, Padua, Italy; 5 Data Science Unit, Department of Epidemiology and Data Science, Fondazione IRCCS Istituto Nazionale dei Tumori, Milano, Italy; 6 Elekta SA, Boulogne-Billancourt, France; 7 Medical Physics Unit, A.O.U. Città Della Salute e Della Scienza di Torino, Torino, Italy

**Keywords:** radiomics, machine learning, artificial intelligence, guidelines & recommendations, medical imaging

## Abstract

**Introduction:**

Radiomics aims to develop image-based biomarkers by combining quantitative analysis of medical images with artificial intelligence (AI) through a robust, reproducible pipeline. Scientific societies, task groups, and consortia have published several guidelines to help researchers design robust radiomics studies. This review summarizes existing guidelines, recommendations, and regulations for designing radiomics studies that can lead to clinically adoptable biomarkers.

**Methods:**

Relevant articles were identified through a PubMed systematic review using “radiomics” and “guideline” as keywords. Of 314 retrieved papers, after screening 99 articles were deemed relevant for extracting recommendations on developing image-based biomarkers. Additional guidelines were searched by the authors.

**Results:**

We can synthesize the systematic review in the following high consensus recommendations divided into five major areas: a) Study Design: Carefully define the study rationale, objectives, and outcomes, ensuring the dataset is of adequate size and quality; b) Data Workflow: Use standardized protocols for image acquisition, reconstruction, preprocessing, and feature extraction—following IBSI guidelines where applicable; c) Model Development and Validation: Follow best practices for model development, including prevention of data leakage, dimensionality reduction, strategies to enhance model interpretability, and establish biological plausibility; d) Transparency and Reproducibility: Publish results with sufficient methodological details to ensure rigor and generalizability and promote open science by sharing codes and data; e) Quality and compliance: Evaluate study compliance with relevant guidelines and regulations using appropriate quality metrics.

**Conclusion:**

Radiomics promises to offer clinically useful imaging biomarkers and can represent a significant step in personalized medicine. In the present systematic review we identified five key guidelines and regulations developed in recent years, specifically for radiomics or AI, that can guide the research community in designing and conducting radiomic studies that result in an imaging biomarker suitable for clinical practice.

## Keypoints

• Study design: Carefully define the study rationale, objectives, and outcomes, ensuring the dataset is of adequate size and quality.
• Data Workflow: Use standardized protocols for image acquisition, reconstruction, preprocessing, and feature extraction—following IBSI guidelines where applicable.
• Model development and validation: Follow best practices for model development, including prevention of data leakage, dimensionality reduction, strategies to enhance model interpretability, and establish biological plausibility.
• Transparency and Reproducibility: Publish results with sufficient methodological details to ensure rigor and generalizability and promote open science by sharing codes and data.
• Quality and Compliance: Evaluate study compliance with relevant guidelines and regulations using appropriate quality metrics.

## 1 Introduction

Radiomics applies quantitative analysis to medical images using high throughput calculations to extract mathematical descriptors called radiomic features (1–3), assuming that images encode biological processes which can be captured through voxel relationships, intensity distributions, and texture (1, 2). Thus, images are converted into data that can be mined using artificial intelligence (AI) techniques to study correlations with diagnostic or clinical endpoints. The aim is to identify features, or a radiomic signature (a combination of features), that could be used to diagnose or predict patient outcomes (4) or to tailor precise medical applications to individual patients (5), thereby helping shift from population-based approaches to truly individualized medicine.

Driven by the potential to enhance diagnostic accuracy and support clinical decision-making, interest in radiomics and in AI applied to medical imaging—fields that are closely interconnected—has been steadily increasing (1–3, 6, 7). However, radiomics has faced challenges in building clinically meaningful imaging biomarkers during its progress, stemming from the time-consuming data collection which involves manual segmentation (8), the multiple sources of variability impacting generalizability and reproducibility, and the large number of feature extracted, often redundant or irrelevant for the task at hand, bringing problems of overfitting. As a consequence of these challenges, no published study has yet prospectively implemented radiomic models as a routine clinical decision-support tool (9).

To address the need for more generalizable results, it is necessary to standardize the multistep process of radiomic analysis, encompassing image acquisition, feature calculation, and machine learning (ML) (1, 10). Equally crucial is defining the safety and efficacy boundaries of any proposed radiomic approach (11). As a result, major efforts have focused on creating recommendations for robust analysis, validation, and data sharing. The purpose of this review was to summarize the existing indications, guidelines, and regulations that radiomics researchers—particularly within the medical physics community—must follow to develop clinically usable imaging biomarkers.

## 2 Systematic review

**Figure 1:**
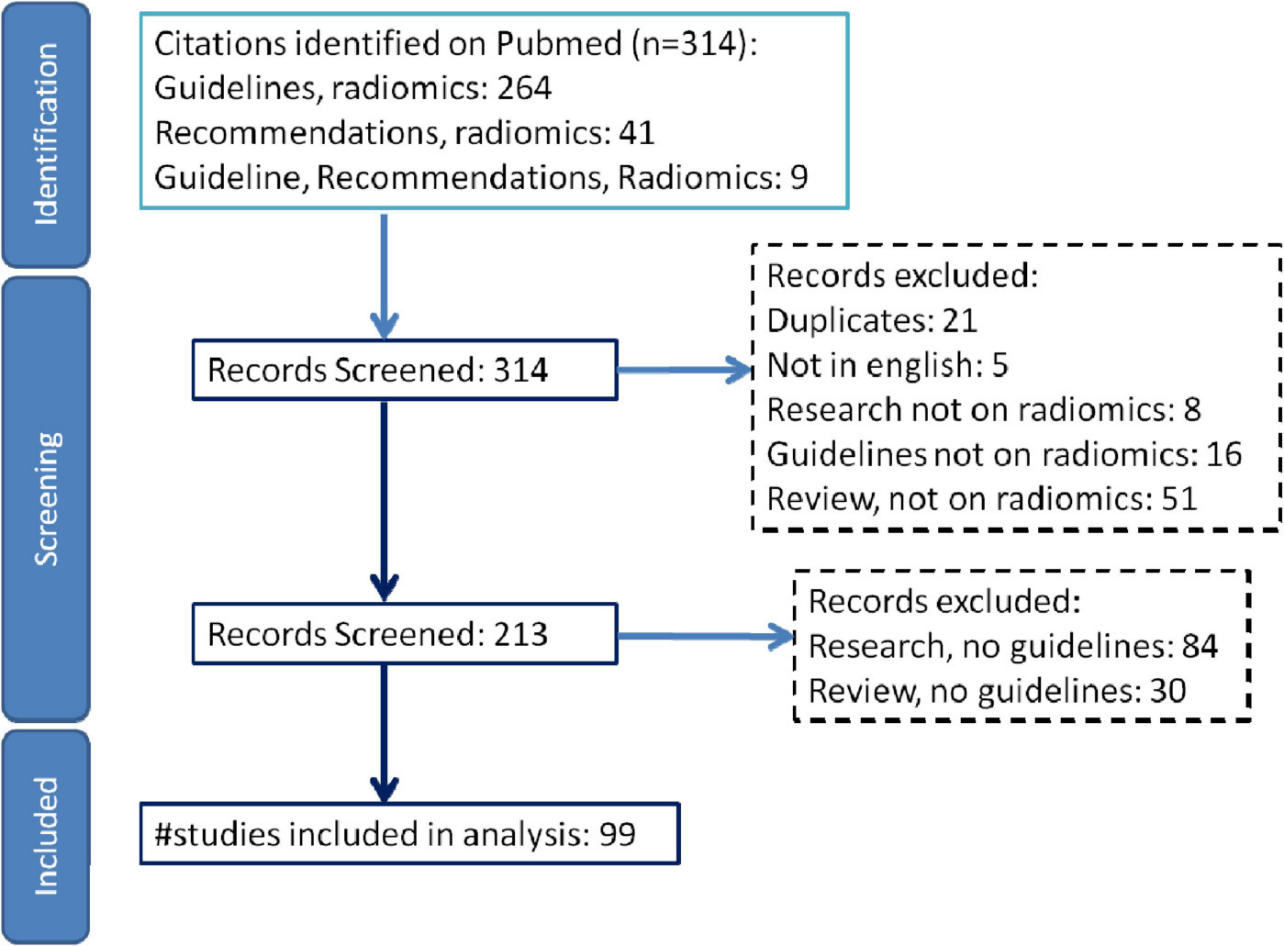
Flow diagram visually summarizing the screening process according to PRISMA statement (13).

A systematic review was performed searching Pubmed for studies providing radiomics guidelines using the keywords “radiomics” and “guidelines”. The results were screened using the Rayyan web-based tool (12), yielding 312 papers. After removing duplicates and studies that provided no recommendations, 99 papers were deemed relevant (Figure 1). The recommendations extracted are summarized in Table 1, where they are grouped in five major workflow areas-from defining the research question to evaluating the clinical utility of the radiomic tool.

**Table 1:** Guidelines on radiomics summarized from the systematic review.

Area	Methods	Key citations
Study Design	- Describe the research question and study rationale, including the hypothesis, justification for using radiomics (14–20), task type (e.g., diagnostic or prognostic) (15, 21), intended role of the radiomics model in clinical decision-making, and potential patient benefits (20, 22–24). - Clearly define the endpoint/outcome measure the radiomic tool should predict (6, 14, 20, 25, 26) in reliable, measurable terms (27, 28). - Describe the study design (e.g., retrospective, prospective, case-control) (14, 21, 29–31). Prefer prospective, multicenter studies (17, 30, 32–44). For retrospective studies, specify data sources, completed studies, databases, or repositories (14, 24). Report eligibility criteria and selection method (random or consecutive) (14, 18, 20, 22, 32). - Ensure the study cohort is representative and of sufficient quality, with diversity and balance (e.g., gender, ethnicity), adequate size, and statistical power (14, 15, 18–20, 22, 30, 34, 39, 45–48). Describe how these were determined (14, 20, 22), e.g., events-per-variable (16, 20), patients-per-feature, learning curves or other approaches (15, 20, 21). Report sample homogeneity (19), balance (15), attrition, censoring, missing data (15, 49), and potential biases (18, 19, 34, 46) evaluated using tools such as PROBAST (50–52). - Obtain committee approval (14, 53, 54) and register study in trial database (14, 21, 44, 55).	CLEAR (14), CLAIM (22) CRUK/EORTC (29), PROBAST (52)
Data Workflow	- Follow standardized protocols for image acquisition and reconstruction (20, 21, 24, 26, 38) e.g., QIBA/QIN for MRI (56) or EARL for nuclear medicine (57) to reduce confounding factors (16, 20, 21, 32, 40, 44, 45, 53, 58–61), while less rigid protocols may better reflect real-world data (15, 48, 62). - Report full imaging protocol, including scanner vendor, acquisition/reconstruction parameters (32, 63) filters, acquisition matrix dimension and resolution, modality specific details (15, 20, 30, 33, 34, 42, 43, 64–69), quality assurance procedures (20, 33, 43). - Define in advance demographic and clinical data relevant to the outcome (18, 30). Use standardised methods for clinical data (e.g., cancer stage, treatment), and ensure adequate follow-up (30, 70). Fully describe the diagnostic protocol (38, 71), including data deidentification (22, 47). - Store image data in a repository in DICOM lossless format (15, 48), with ROIs in DICOM RT (72). Curate for errors and missing data (48), deidentified according to a disclosed protocol (73). - Preprocess images to optimize quality (18, 24, 26, 32, 66) using well-specified steps consistent with IBSI (25, 26, 43, 69, 74, 75): artifacts removal (16, 76), harmonisation (16, 18), intensity normalization (77, 78), bone removal, motion correction, thresholding (14, 15, 22, 33, 43, 65), and noise reduction (15, 33, 66). Disclose preprocessing steps and software (14, 18, 69). Report image conversion [e.g., PET counts to SUV (14, 79), ADC maps from raw diffusion MRI (33, 43, 65)]. Resample to a common isotropic voxel size (18), reporting parameters (14, 15, 30, 33, 42, 43, 65, 78, 80) and interpolation method (14, 33, 65). - Standardize and describe ROI segmentation criteria (14, 15, 32, 36, 59). If multimodal imaging is used, report registration (14, 30, 33, 43, 65, 71). Report software (14) and methods to interpolate binary masks from ROIs, including partial-volume management if applicable (33, 65). - Describe the segmentation process. Number of experts (16, 33, 38, 48, 81), and expertise (14, 33, 43, 65) of readers reported with inter-reader variability assessed (15, 16, 33, 43, 82) by ICC (14, 42, 54, 63, 78, 83, 84). Indicate whether manual, semiautomatic (18, 30, 33) of fully automated methods are used (15, 20, 33, 82). The latter should be reviewed (15) or formally evaluated (32). - Describe preprocessing before feature extraction (14), e.g., intensity discretization (fixed bin number or width) (33, 78), and histogram equalization (33, 42, 43, 65, 80). Preprocessing steps should follow IBSI (18, 78) with parameters reported (24, 30, 85) and rationale provided (30). - Provide a standardized, well-described workflow for feature extraction (21, 32, 66, 78), adhering to IBSI methodology and nomenclature (20, 24, 25, 30, 33, 42, 43, 49, 65, 69, 86). Use IBSI-benchmarked software (18, 32, 68, 69, 85, 87–91), reporting software type and version (33, 43, 65, 80, 85). - Report the number, classes, and names of features (14, 21, 24, 43, 67), calculation settings (e.g., voxel connectivity, 2D or 3D (14, 33, 43, 65), and use a standard lexicon (92, 93). - Describe post-processing of features, e.g., scaling/normalization to a common range and center (14, 20, 30, 94), and harmonisation across protocols, devices, or institutions (14, 18, 20, 95, 96).	STARD-AI (81). CLAIM (22) ESR (97) IBSI (65) CLEAR: (14)
Model Development and Validation	- Perform proper preprocessing (14, 24), e.g., handle outliers and missing values (14, 20, 82), adjust for confounders (14), and address data imbalance (e.g., by SMOTE) (14, 30, 39). - Apply dimension reduction techniques (14, 39, 53, 90, 98) to minimize overfitting risk (16, 20, 21, 24, 32, 34, 38, 42, 86, 94). - Remove unstable or unreliable features (14, 21, 32, 33, 43, 78) e.g., from test-retest analysis (14, 17, 26, 33, 42) or variability studies on slice thickness (99) discretization/resampling (100), reconstruction filters (21), or other imaging settings (24, 33, 43, 70, 86) including phantom study (21, 26, 35). Report reproducibility metrics (20) such as ICC (14, 78, 83) or accuracy changes (100). - Evaluate feature collinearity using Pearson, Spearman, Euclidean distance (30, 33, 90) and eliminate highly intercorrelated features (14, 33, 66, 90). - Remove features not correlated with outcome after univariable analysis (14, 20, 33) e.g., ANOVA or Kruskal Wallis (67), Chi-squared for two classes, Mann–Whitney for non-normal two class data (67). - Apply algorithm-based dimensionality reduction techniques (30), such as principal component analysis (14, 20), or Algorithm-based Feature selection (14) such as clustering, LASSO (54, 71, 90, 101), ML-based feature selection (14, 16, 20, 21) with properly tuned parameters (90). - Specify how the final number of features was chosen, e.g. ≤ 10 features per instance (14), ≤ 50 weights in deep learning (102) or by testing performance vs. number of features (103). - Split data into training and validation before the training process to prevent leakage (16, 18, 19, 43). Report split criteria (e.g., random, by institution, proportion) (14), and indicate event distribution and class imbalance (39). - Select ML models appropriately (67, 94, 104) e.g.by comparing multiple approaches (18, 21), or preferring explainable models (61, 102). Choose the final model with criteria such as Akaike Information Criterion (14). Report details: architecture, software and version (14, 19, 20, 22, 54), training process, and hyperparameters (14, 30). - Report calibration plot and metrics (14, 18, 20, 32, 44) e.g., actual vs. predicted risk, and Hosmer-Lemeshow statistical test (21, 42, 54). Use appropriate metrics (32): accuracy, sensitivity, specificity, AUROC, (18, 21, 51). Define cutoffs (21, 70) e.g., with Youden index (79), for classification (20); R^2^ or adjusted R2 for regression (20). Describe metrics pitfalls (14, 51) and assess uncertainty (e.g., confidence intervals) (19, 32, 51). - Assess associations between variables and clinical data (19, 30, 31, 66), correct for multiple testing (e.g., Bonferroni, Benjamini–Hochberg) (33, 43), and use nomograms to enhance interpretability (105, 106). - Integrate non-radiomic variables such as clinical/demographic data (14, 20, 30, 38, 61, 94, 107), therapies (108), radiologist assessments (e.g., PIRADS) (14, 16, 33, 43, 44, 106), or sequencing data (radiogenomics) (62, 66–68). - Perform end-to-end evaluation of model performance in validation and test sets (14, 18, 29, 32, 33, 43, 44, 104) using internal methods (bootstrap, k-fold, repeated k-fold, random split) (46) or nested CV when no external data are available (14, 20, 21, 31–35, 43). Describing the resampling technique (14). - Independently validate (18, 29, 32, 44, 75, 109–111), using data from hospitals other than those that developed the model (31, 46), ideally from different regions (43) and from a public database (82).	EANM/SNMMI (18), CLEAR (14), ESR (97)
Transparency and Reproducibility	- Write paper following reporting guidelines (17, 80, 112) such as CLAIM (32, 113) or TRIPOD (6, 17, 87, 111, 114), STARD-AI (81). Include a clear title, abstract and introduction (22), sufficient background (22), study design, and a well described pipeline (105), e.g., with a flowchart (14). Present results clearly (14), including feature characteristics and model performance. In the discussion cover findings, prior work, implications, strengths/limitations, validity and generalizability, (14, 19, 22, 26, 49, 69) and provide clear conclusions (22). - Share study code (36, 43, 85, 98) as easy-to-run, organized scripts with instructions (14, 33, 53) following FAIR principles (Findable, Accessible, Interoperable, and Reusable data) (18). - Share image data with segmentations/annotations, radiomic and clinical variables (14, 17, 18, 21, 25, 30, 36, 42, 53, 67, 114), and other datasets e.g., test-retest images (21). Provide a clear description of ground truth data e.g., annotation tools (22).	TRIPOD (115) FAIR principles (116) STARD-AI (81)
Quality and Compliance	- Assess study compliance with relevant guidelines and report standardized quality metrics e.g., RQS (21, 25, 35, 38, 41, 42, 49, 58, 61, 68, 69, 75, 80, 85–87, 98, 101, 106, 114, 117–122), RANDAM (20), CLEAR (14, 69, 80) CLAIM (22, 47), STARD for diagnostic studies (112), METRICS (32), PET-specific checklist (123), If reproducibility is studied, follow appropriate methodology (124). - Evaluate risk of bias in patient selection, applicability, and endpoint measurement using QUADAS-2 (18, 35, 58, 61, 87, 98, 101, 109, 114, 118, 121) or QUIPS (25). - Report radiomic model development in line with TRIPOD guidelines (17, 18, 26, 30, 32, 42, 43, 49, 68, 75, 87, 110, 111, 114, 117, 120). - Interpret black-box models carefully to avoid conflating correlation with causation, introducing confirmation bias, or focusing on irrelevant data (30, 68). Use interpretability tools such as class activation maps, feature importance (14, 22), Shapley additive explanations (102, 120), or failure analysis of misclassified cases (47). Correlate radiomic with non-radiomic features where relevant (21, 33, 54). - Establish biological plausibility (20, 29) by assessing relationships between radiomic tumor phenotypes and underlying microscopic biology (33, 42, 43, 46, 60, 93, 97). - Discuss potential clinical applications of the radiomics test (17, 21, 22, 24, 33, 44, 111), or directly evaluate its performance in the intended use context (24). - Compare the radiomics-based model to a clearly defined reference or gold standard (14, 20–22, 30–33), explaining the choice (14, 22) and limitations of the chosen reference standard (14). - Evaluate the added value of radiomics both statistically (e.g., significance of AUC increase) (33, 43), and in terms of cost effectiveness (29), e.g., QALY (17, 21, 35, 42, 46, 98).	CLAIM (22), CLEAR (14), METRICS (32) TRIPOD (115)

### 2.1 Designing an image biomarker study

Seminal radiomics papers hypothesized that distinctive image-derived features could help predict prognosis and therapeutic response across cancer types (125, 126), thereby identifying imaging biomarkers. According to the FDA and NIH Biomarker Working Group (29), a biomarker is “an indicator of normal biological processes, pathogenic processes, or responses to an exposure or intervention”. This definition includes radiographic characteristics, thus explicitly including features that can be extracted from medical images (127). These can be semantic or agnostic. Semantic features are qualitative descriptors assessed visually by radiologists, such as size, shape, location, vascularity, spiculation, or necrosis. Radiomic features are agnostic features, meaning that they are mathematically extracted imaging descriptors (128). Imaging biomarkers are non-invasive and allow comprehensive evaluation of the 3D tumor landscape through extraction of relevant imaging data. Examples of quantitative imaging biomarkers already used in the clinic include standardized uptake value (SUV) from PET (57) for tumor glycolytic activity, longest tumor diameter for assessing response in RECIST (119), and splenic volume from CT or ultrasound (29, 129–131).

Cancer Research UK (CRUK) and the European Organisation for Research and Treatment of Cancer (EORTC) produced 14 key recommendations for achieving the clinical translation of imaging biomarkers into clinical practice (29). These guidelines, along with those identified in our systematic review, begin with defining the research question and rationale for using radiomics (20).

The selection of the primary “endpoint” or “outcome measure” has considerable influence on the reliability and interpretability of radiomics studies. To maximize informativeness, primary endpoints should be well defined, reliable, measurable, and interpretable (14, 20, 21, 23–26, 28), and ideally based on gold-standard methods (27). Also, the possible role of the radiomics model in clinical decision-making should be defined at this stage (20, 23, 24).

When designing a radiomic study, the type of biomarker under investigation determines study design (6). A *predictive biomarker* stratifies patients more likely to respond to a therapeutic agent, e.g., achieving local control (103, 132). A *prognostic biomarker* is used to identify the likelihood of a clinical event, which could be recurrence, progression, survival or a side effect. Examples include the assessment of the probability of survival in patients with a specific cancer type (133). A *diagnostic biomarker* can detect a disease or a specific subtype e.g., the presence of a gene mutation (134). Delta-radiomics, where radiomic features monitor tumor changes during therapy (135), is an example of *monitoring biomarker*.

Biomarker assessment should be accurate, reproducible, and feasible over time (136). When biomarkers also reflect the effect of disease-specific treatments they are termed 'surrogate endpoints' (137), characterized by a biological link to the true endpoint, proven prognostic value, and evidence that treatment effects on the surrogate reflect clinical outcomes (138).

At this stage, inclusion and exclusion criteria, as well as the selection process (random or consecutive), should be defined (14, 18, 20, 22, 32), ensuring that study participants are representative of the intended patient population and that there is appropriate diversity and balance of characteristics (18–20, 30, 48).

A size-estimation analysis should also be carried out to determine the sample size needed to answer the research question (20). Radiomic studies are often monocentric and retrospective, partly due to challenges in collecting and securely sharing prospective patient data (139). In retrospective radiomic studies, imaging protocols (acquisition and reconstruction) are often uncontrolled or non-standardized (8). Such studies are also prone to bias, as they depend on existing medical records and offer limited control over participant selection (140).

### 2.2 Data collection

The radiomics workflow begins with image acquisition (1, 8, 128). Images should be acquired at the appropriate time relative to the endpoint and in accordance with standardized, well-documented protocols or consensus guidelines for acquisition and reconstruction (20, 21, 24, 26, 38), such as those from the Quantitative Imaging Biomarkers Alliance (QIBA) for MRI (56) or European Association of Nuclear Medicine (EANM) for nuclear medicine (57). Image acquisition guidelines help remove confounding factors (16, 21, 32, 40, 44, 45, 59–61) that may obscure correlations between tumor biology and imaging data (20, 53, 58). However, less rigid protocols may sometimes produce radiomic models that better reflect real-world scenarios (15, 48, 62). Imaging protocol details should be disclosed, including scanner vendor and acquisition and reconstruction parameters (32, 63), such as filters, field of view, acquisition matrix dimensions and resolution, CT x-ray energy and exposure, MRI sequence, PET acquisition time, and administered activity (15, 20, 30, 33, 34, 42, 43, 64–69).

Segmentation also critically affects the radiomics workflow. In fact, if performed manually, it affects radiomic features by inter-observer variability of contours (8, 141). Unfortunately, there is no consensus on how to delineate tumor regions specifically for radiomic analysis (123). In order to reduce the variability in radiomic feature values because of the Volume of Interest (VOI) definition, it is recommended to define a common rule among the patient dataset for segmenting lesions (142). Semi-automated or fully automated segmentation, also made possible by deep neural networks, can reduce inter-user variability of contours and improve the stability of radiomic features (8, 143, 144). Ground truth description and the definition of the annotation approach are of particular importance, specifying the number and expertise of experts involved, and the methodology adopted to get the final consensus. In the case of multiple experts involved, to reduce annotation biases, the intra- and inter-observer variability (145), using the intraclass correlation coefficient (146) or the Cohen's kappa (147), is encouraged.

Images should be exported as Digital Imaging and Communications in Medicine (DICOM) files in a lossless format (15), including regions of interest (ROIs) in DICOM RT structure format (72). The image de-identification/anonymization protocol should be disclosed (73). After collecting imaging and clinical data, these should be reviewed for errors and missing entries (48) and stored in a curated repository for analysis by a competent data curator (15, 48). Clinical data should be collected using a standardized methodology (e.g., cancer stage, treatment details, and adequate follow-up) (30, 70). The diagnostic protocol should be also fully described, including the imaging modality used, the timing of acquisitions if multiple time points are involved and the data de-identification/anonymization process (38, 47, 71).

### 2.3 Preprocessing and feature extraction

**Figure 2:**
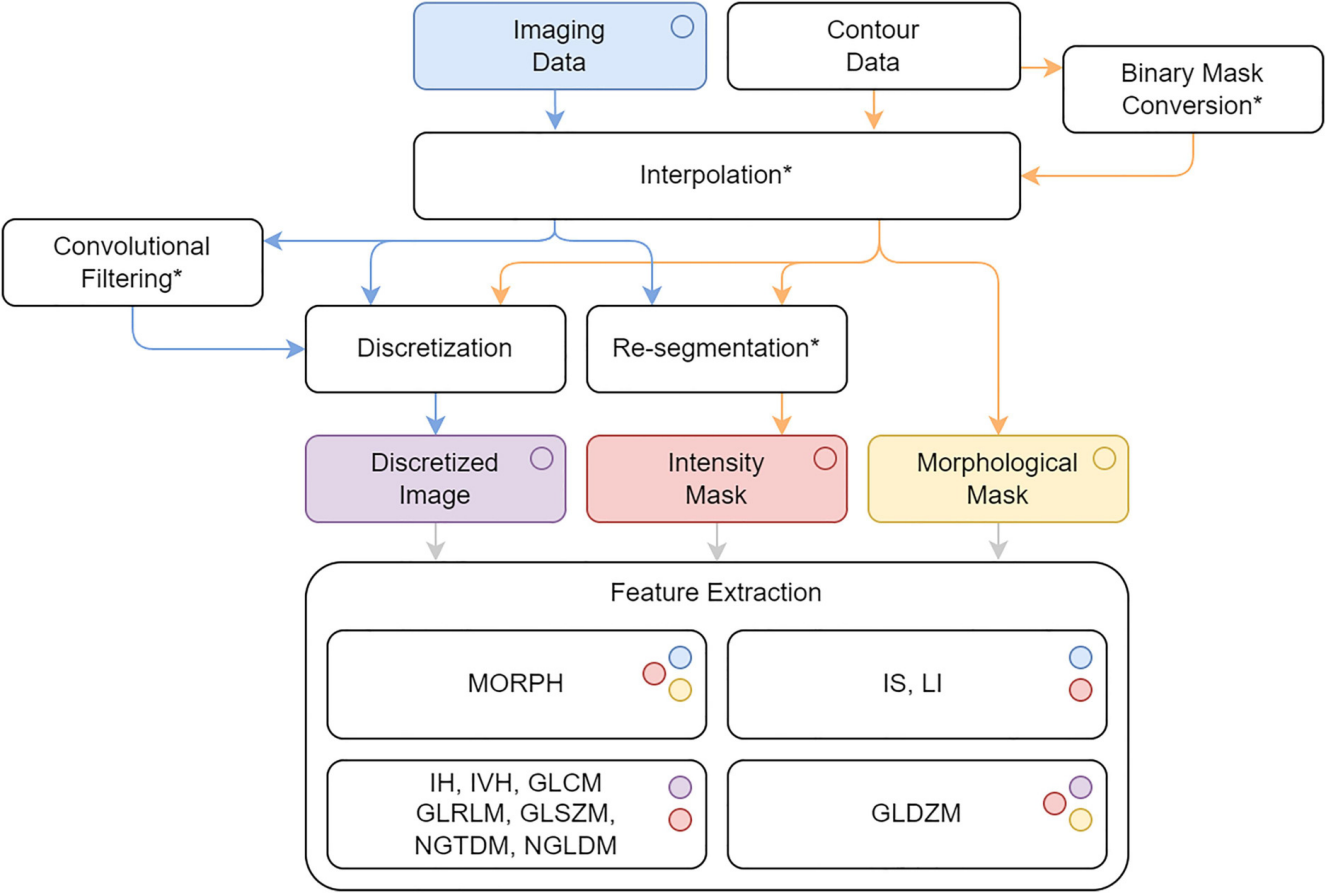
Radiomic workflow as standardized by IBSI. *optional steps. MORPH: morphology, LI: local intensity, IS: intensity-based statistics, IH: intensity histogram, IVH: intensity-volume histogram, GLCM: grey level co-occurrence matrix, GLRLM: grey level run length matrix, GLSZM: grey level size zone matrix, GLDZM: grey level distance zone matrix, NGTDM: neighborhood grey tone difference matrix, NGLDM: neighboring grey level dependence matrix.

An international consortium, the Image Biomarker Standardization Initiative (IBSI) (148), standardized the overall workflow of radiomic feature extraction (depicted in Figure 2), providing guidelines for each of its steps (Table 2), standard nomenclature and mathematical formulations of features (149), and standardized protocols for filtering in radiomics (150).

**Table 2:** IBSI recommendations regarding both pre-processing and feature calculation.

Task	IBSI recommendations	Rationale
Binary mask conversion	The crossing number algorithm is advised for determining if a point belongs to a 2D polygon.	The crossing number algorithm is computationally simple, robust, and widely used. It reliably determines point-in-polygon membership even in the presence of holes and disconnected regions.
Image & binary mask interpolation	Prefer 3D resampling for 3D images. When voxel size is not isotropic, 2D slice-by-slice interpolation may be preferable. Perform mask interpolation using nearest-neighbor or trilinear methods. When aligning the voxel grid, the “align grid centers” method is recommended as it is more robust and requires fewer parameters.	2D interpolation avoids axial blurring and loss of information when spacing between slices is significantly larger than the desired voxel size. If the resulting voxel dimensions are not isotropic, features should only be calculated with in-plane aggregation methods.
Mask interpolation	Mask interpolation should be performed using nearest-neighbour or trilinear interpolation methods; in the latter case, values must be binarised using a partial-volume threshold, typically set to 0.5.	Nearest-neighbour preserves the binary nature of masks but produces blockier edges, while trilinear interpolation yields smoother contours but introduces fractional volumes that must be thresholded. Using a 0.5 threshold retains voxels that are predominantly inside the ROI.
Re-segmentation & discretization	Select appropriate re-segmentation and discretization methods. Both FBN and FBS can be used, but if re-segmentation is not applied or the image is in arbitrary units, use FBN only.	FBS preserves a direct link to the physical intensity scale but becomes unreliable when intensities lack defined units. To ensure consistency across samples, FBS should always be applied in conjunction with a specified lower bound of the re-segmentation range.
Feature families computation	For morphological features, use the unit of length defined in the metadata, ensuring consistency across all cohort data. For intensity statistics (IS), intensity histogram (IH), and intensity–volume histogram (IVH) features, use a 3D volume aggregation approach. For the IVH family, perform discretization separately with a bin width small relative to the ROI intensity range (smaller than that used for other feature families).	Averaging IS, IH and IVH features across slice is rare, and not recommended. For IVH features, dense discretization is recommended when necessary. If the image intensities are already discrete, no further discretization is required. For continuous intensity values, discretization should be applied using a bin width small relative to the ROI intensity range to avoid coarse approximations.

Preprocessing aims to reduce image noise, enhance image quality, and harmonize images before feature computation. It can mitigate protocol-related differences, for example by interpolating datasets to a common grid size (151), and includes converting gray levels to meaningful physical units such as SUV in PET or Hounsfield Units in CT (103). Filtering—typically via convolution kernels—can highlight image characteristics such as edges and textures; common filters include wavelets, Gaussian smoothing, and Laplacian edge-enhancing filters, often applied in combination (152, 153). Gray-level discretization into fixed numbers of bins or fixed bin widths is essential for texture analysis, as most matrices require integer values (154), while voxel resampling to a common isotropic size is necessary for certain feature calculations (155). Preprocessing steps should comply with IBSI guidelines (18, 78) and be fully documented, including choices such as intensity discretization strategies [e.g., fixed bin number or fixed bin width such as 25 HU/bin (33, 78)] and histogram equalization (33, 42, 43, 65, 80). When applicable, the rationale for the chosen discretization should be provided—for instance, using 64 bins within a standardized SUV range of 0–20 for PET imaging (24, 30, 85).

Feature extraction is the crucial step in which quantitative features are computed from the ROI, capturing its shape (e.g., sphericity, compactness), statistical (e.g., intensity range, contrast) and textural (e.g., heterogeneity, homogeneity) properties. In order to provide standardized results, a radiomics study should adhere to the IBSI methodology and nomenclature regarding the feature extraction pipeline (20, 24, 25, 30, 33, 42, 43, 49, 65, 69, 86), and the adopted workflow for feature extraction should be well described (43, 65).

Many radiomic software tools are available for commonly employed programming languages, including Python, R, and MATLAB, offering a range of functionalities, from image pre-processing to image segmentation and statistical analysis. Some commonly used and freely available packages include MIRP (156), S-IBEX (157), LIFEx (158), SERA (258), RaCaT (159), ROdiomiX (160), MITK Phenotyping (161), MODDICOM (162), PyRadiomics (163), RadiomiCRO (164).

Radiomic software should comply with the IBSI (18, 32, 68, 69, 85, 87–91). To comply with the IBSI, a software should provide the 169 IBSI-standardized features grouped in 11 feature families, two re-segmentation algorithms (range re-segmentation and outlier filtering), two discretization methods: fixed bin number (FBN) and fixed bin size (FBS) and nine different aggregation methods (e.g., 2D:avg, 2D:mrg, 3D:avg). The software type and version of code used for computation of features should be described (33, 43, 65, 80, 85).

Harmonization is the reduction of variability among radiomic features due to differences in imaging devices, acquisition parameters, reconstruction methods or other factors that may differ among centers (29). Mali et al. (165) identified two domains for harmonization, the image and feature domains. The image domain operates on differences generated by technical parameters used in image acquisition. Image harmonization can rely on digital phantoms (99, 154, 166–182). In feature domain harmonization, a feature normalization is performed resulting in a common range of features. Approaches to mitigate feature variance include residual harmonization (183, 184) and ComBat, originally developed in genomics (185) to suppress non-biologic sources of variance in multicenter studies, and later widely applied to radiomics (4, 184, 186–189), for its low computational burden and open-source availability (187). ComBat has certain limitations, primarily due to its assumption that data from each center follow a normal distribution—an assumption that may not always hold true. Moreover, it does not address collinearity, i.e., feature variations that are simultaneously influenced by both the investigated effect and technical parameters. The modified ComBat (M-ComBat) assumes one center as a reference for the others, allowing new centers to be added incrementally without repeated recalibration. Bootstrap ComBat (B-ComBat and BM-ComBat) employs Monte Carlo resampling to obtain multiple estimates, thereby improving robustness (190). Alternatively, Bertolini et al. proposed to use the grey level in an organ, e.g., healthy lung tissue in CT, as a reference level to compare and adjust values among centres (191).

### 2.4 Building a radiomic model

Like other “omics” fields, radiomics involves many variables and complex, non-linear relationships, making machine learning (ML) the predominant technique for analysis (3, 6, 192). ML requires a training phase, where it analyses a set of data whose endpoint is known, builds a model for classification or prediction and measures its performance (193). Pioneering works on radiomics focused mostly on assigning a categorical output variable, such as whether or not a disease has recurred, or whether the patient is alive beyond a certain time threshold (164). Some endpoints can also be modeled as regression tasks, such as predicting overall survival (194, 195) using survival times and relative risks (196, 197).

It is crucial to describe in detail the structure of the model, from preprocessing to the ML pipeline, including software programs and libraries used. It is also essential to specify the technical, clinical or ethical guidelines that were adopted. The most widely used ML algorithms include decision trees (198), ensembles of decision trees (199), support vector machines (200), and neural networks (201). Choosing a suitable ML model may involve comparing multiple approaches (18, 21, 67, 94, 104) or favoring more interpretable models such as decision trees or logistic regression, especially with small datasets (102). The final model should be selected using an established criterion, such as the Akaike Information Criterion (AIC) or similar methods (14). The type of model used, along with its architecture, software, and version, should be clearly specified (14, 19, 20, 22, 54).

Radiomic studies often use feature selection algorithms to remove non-predictive or redundant variables and identify the optimal subset from the large number of extracted features (202, 203). Broadly, feature selection methods fall into three categories: (i) Embedded methods, where the learning algorithm itself determines the optimal subset of features; (ii) Filter methods**,** which select or discard features prior to learning based on their relevance; and (iii) Wrapper methods, which assess model performance across different feature subsets to identify the most suitable set (14, 16, 20, 21, 54, 71, 90, 101). The final number of features can be set using rules of thumb, e.g., ≤10 features per instance (14), ≤50 weights in deep learning (102) or by selecting the number that yields optimal performance (103).

The tuning of the parameters in the ML pipeline should be rigorously carried out on the training set to avoid bias. The No Free Lunch Theorem states that no algorithm can be optimally tuned for all problems (204). Moreover, tuned parameters often perform no better than default values reported in the literature (205). The tuning strategy needs to be fully described, along with the number of iterations and range of hyperparameters (14, 30).

Choosing appropriate performance metrics is crucial for reliable model development and evaluation. For classification tasks, performance is commonly measured using metrics derived from the confusion matrix, such as accuracy, specificity, sensitivity, precision and recall. However, class imbalance—where one class has far fewer instances—is frequent in radiomics and can reduce performance on minority classes (206). Metrics such as the F1-score, balanced accuracy, and the geometric mean of accuracy account for imbalance (207). For models that output posterior probabilities, performance can be assessed with ROC curves and summarized by the area under the curve (AUROC). AUROC reflects the probability that a positive instance is ranked above a negative one, equivalent to the Wilcoxon rank-sum test (208). Despite the usefulness of the AUROC, ROC curves should still be reported, since models with the same AUC can perform differently in specific regions of the ROC space (208). The area under the Precision-Recall curve allows assessment of model performance on the minority class, independent of the majority class (209, 210).

The metrics for regression, e.g., predicting the time of the occurrence of metastasis, include the Mean Absolute Error (MAE), the Mean Squared Error (MSE), the Relative Absolute Error (RAE), the Root Mean Square Error (RMSE), and the Relative Squared Error (RSE): for all of them, the more their values are close to zero, the better the performance. R^2^ is used only for those methods that employ linear regression while adjusted R^2^ also accounts for the goodness of fit, sample size, and the number of predictors used (211). To overcome the issue of class imbalance, re-sampling can be applied, of which over-sampling has been proven more effective in ML (212, 213). Commonly used methods for oversampling minority class include the Synthetic Minority Over-sampling Technique (SMOTE) (14, 30, 39). Finally, robust statistical assessment of results is essential. There are many statistical tests to be considered and one of the first choices is whether to use pairwise or multiple tests (214, 215).

Because ML models often function as “black boxes,” interpreting their outputs is essential to avoid conflating correlation with causation, reinforcing bias, or emphasizing irrelevant data (30, 68). Beyond favoring inherently interpretable models (61), interpretability can be enhanced through class activation maps, feature importance analyses, e.g., by Shapley additive explanations (14, 22, 102, 120), and by examining misclassified cases (47).

### 2.5 Dissemination of results

When publishing a study on radiomics, the primary goal should be to enable other researchers to reproduce its results. The lack of reproducibility, mainly due to insufficient description of data source and methods in the manuscripts (11), is the Achilles' heel of radiomics (20). Koçak et al. identified the lack of consensus in many steps of the radiomic pipeline, stressing the importance of transparently reporting key information (216), and later produced an extensive list of 22 best practices for the steps of pre-modeling, modeling, and post-modeling (217). Several proposals have addressed the necessary quality checklists to ensure rigor, quality, and generalizability of radiomic studies and assess their quality, starting with the radiomic quality score (RQS) (21), the METRICS quality checklist (32) and the PET radiomics checklist (123). The Radiomic Analysis and Data Modeling (RANDAM) checklist (20) contains five main components to be used in a radiomic study, ensuring that a model is exactly reusable by other researchers. The Standards for Reporting Diagnostic Accuracy (STARD) initiative introduced a flow diagram describing key aspects of diagnostic studies, including patient recruitment, test execution order, and the number of patients undergoing the index test and/or reference standard (112). STARD was recently expanded with the STARD-AI statement (81), specifically tailored to AI-based studies. The CLAIM checklist (22), deriving from STARD, provides additional best-practice recommendations for researchers. Study data quality and diagnostic accuracy can be assessed—particularly regarding patient selection, applicability, and endpoint measurement—using tools such as QUADAS-2 (18, 35, 58, 61, 87, 98, 101, 109, 114, 118, 121) or QUIPS (25).

**Figure 3:**
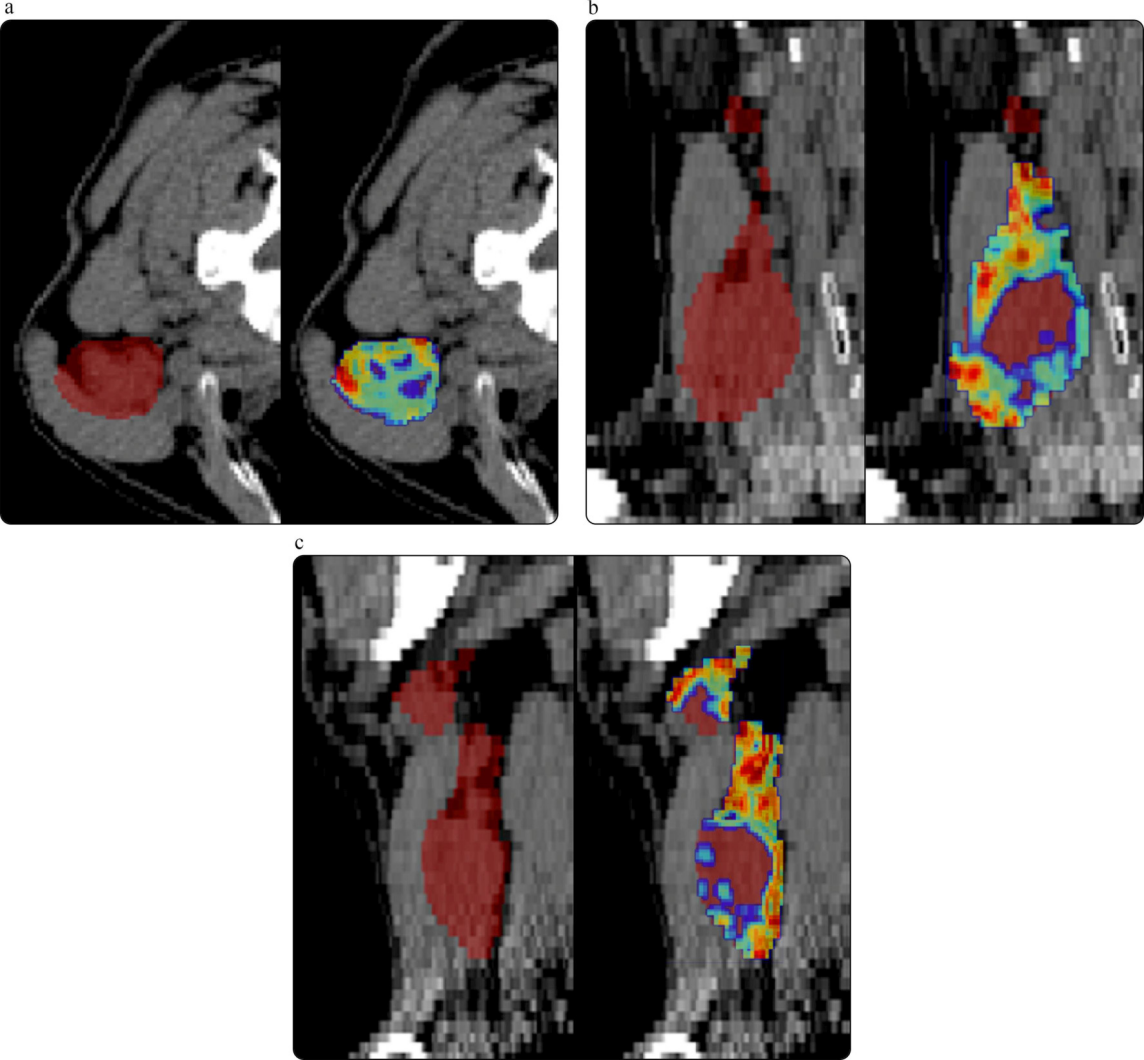
**(a–c)** Example CT scan of a head and neck cancer patient from TCIA, shown with the contoured gross tumor volume (GTV). Side-by-side images display the first-order radiomic feature “entropy” calculated voxel-wise within the GTV using the open-source PyRadiomics package, in axial **(a)**, coronal **(b)**, and sagittal **(c)** views. Feature extraction was performed with PyRadiomics running in the free Google Colab environment (colab.research.google.com).

Authors of radiomic studies are strongly encouraged to deposit the data and/or software used for modeling and analysis in publicly accessible repositories and to provide direct links to these resources. Open data consist of datasets that can be freely used, modified, and shared for any purpose, including external validation. Several online repositories host imaging datasets, most notably The Cancer Imaging Archive (TCIA) maintained by the National Cancer Institute (218), giving the possibility of training or validating new models (219). Open-source refers to software whose source code is freely available to access, modify, and share, ensuring transparency in the methods used to obtain scientific results and enabling others to extend or adapt the code for related research. In addition to open-source tools for radiomic feature extraction, widely used free libraries such as scikit-learn (http://www.scikit-learn.org) can be run within Python (http://www.python.org), a high-level and easy-to-learn programming language. An example of radiomic feature extraction using open-source software on a patient from The Cancer Imaging Archive (TCIA) is shown in Figure 3.

## 3 Validating a radiomics biomarker for clinical use

In this section we will summarize the steps required for the ultimate goal of radiomic biomarker, adoption in clinical routine. Technical validation of a biomarker includes measurements of reproducibility and robustness to ensure that the biomarker can be measured in any geographical location by any operator or equipment, producing comparable results. Reproducibility and robustness should not be used as the only figure of merit to evaluate features. They can still be compared against sensitivity, the ability of a feature to change when the grey-level distribution undergoes a change potentially occurring in a clinical context (146).

Clinical validation is the process of demonstrating the clinical utility of a feature or multiple features embedded in a statistical or ML model, e.g., by association with an endpoint or characteristics of a disease (97). Translation into clinical practice implies the commercialization of the output of the investigational study by a manufacturer, which has to take responsibility and liability for the safety and efficacy of the product. Regulators are tasked to guarantee the product's development and post-marketing of the product according to applicable laws, directives, and derived standards (220).

### 3.1 Repeatability and robustness

Repeatability refers to the precision of measurements performed multiple times in the same subject (*in vitro* or *in vivo*) under identical conditions using the same equipment, software, and operators over a short timeframe (221). Robustness is the ability of features to reflect the biological or clinical characteristics of the tumor or organ without being affected by technical factors, such as different scanners, imaging protocols, feature extraction methods, or operator-dependent procedures like manual segmentation (99, 145), ensuring that a robust feature can distinguish between patient groups even under varying conditions (137). The QIBA initiative suggested methods for measuring in-phantom robustness to change in imaging protocols (222).

Robustness to the image acquisition process has been extensively studied (155, 223–228), examining the effects of different scanning protocols or machines using phantoms specifically designed for CT (167, 171, 229), PET and MRI (8). These studies demonstrated radiomic features dependency on both the acquisition (99, 154, 179–182) and pre-processing parameters (166–178). For improving robustness, IBSI concluded that the same pre-processing parameters should be selected in a multicentric data collection (148). Images should be also free from artifacts in the region of interest, such as those caused by metal implants in CT (133) or motion (15).

Benchmarking evaluates the agreement among radiomics software tools. Compliance with the IBSI nomenclature and feature definitions is assessed by computing over 2,100 feature values using six specific extraction parameter configurations on two IBSI phantoms, and comparing the results with the reference values provided by the initiative. This process has led to the standardization of multiple open- and closed-source radiomic software and the publication of studies assessing feature robustness across different tools (91, 230–232). For example, Bettinelli et al. (91) evaluated seven software programs using the custom ImSURE digital phantoms (89), demonstrating that certain features are influenced by software-specific implementation choices in interpolation, discretization, and aggregation methods.

### 3.2 Clinical validation

The goal of any predictive model is to provide reliable outcome predictions for new patients. Since performance in prospective patients is unknown, it must be estimated from available data and cohorts. Validation measures how predictions perform on out-of-sample data. Radiomic models follow the same validation principles as other predictive models, which can generally be divided into three classes of procedures (115, 233).

Apparent validation assesses performance using the same dataset as model development. It generally results in optimistic biased estimates but may produce unsatisfactory results if the model is applied to new data. It should be limited to proof-of-principle studies on small data sets.

Internal validation, in which training and validation data are drawn from the same dataset through random or non-random splitting, estimates model accuracy for subjects similar to those in the development sample — that is, a dataset with the same feature distribution. It helps correct for overfitting and provides an estimate of optimism when the case mix is unchanged. Here, case mix refers to the distribution of features, both included and not included in the model (234). Internal validation approaches include split-sample (hold-out) methods (115, 233), where data are partitioned into two groups: one for model training (e.g., 50%) and the other for evaluation (e.g., 50%). Random splits can skew predictor and outcome distributions, especially with rare outcomes, and reduce training or validation data, leading to unstable models (115). Non-random splits use fixed criteria, such as enrollment date, center, clinician, or imaging device, which may produce differing case mixes between development and validation sets, providing an initial estimate of model generalizability.

K-fold cross-validation extends the random split-sample approach by dividing the dataset into *k* groups. The model is trained on *k*–1 groups and tested on the remaining group, repeating the process until every subject is used for both training and evaluation. Performance is the average across all k iterations (208).

Bootstrap validation draws random samples with replacement from the original dataset, so some subjects appear multiple times while others are excluded (235). Typically, a large number of bootstrap samples (Z ≥ 1,000) are generated, producing a distribution of performance estimates that allows reporting not only the mean but also measures such as standard error, standard deviation, and quartiles, capturing model uncertainty. Bootstrap validation is particularly effective in high-dimensional settings, where the number of predictors exceeds the sample size, as often occurs in radiomics (236).

External validation tests models on independent datasets from different institutions or populations to ensure generalizability (237) and is feasible only if the training and validation datasets are compatible. It typically involves: (i) validating predictors in the new dataset by comparing risk factor effects, (ii) assessing calibration, i.e., the agreement between observed and predicted outcomes (usually via a calibration plot), and (iii) evaluating discrimination using an appropriate metric. External validation may fail if the case mix differs from the development data, for example, if the predictive effects of features vary across cohorts. Overfitting in the development dataset, especially when many predictors are used with a small sample, can also reduce generalizability. In such cases, the model can be updated or adjusted using the validation dataset, which is encouraged when the validation set is large (234).

Clinical validation of a radiomic tool includes discussing its potential applications in clinical practice (17, 21, 22, 24, 33, 44, 111). Before implementation, the patient benefit must be demonstrated by comparing the tool against a defined reference or gold standard (14, 20–22, 30–33), with justification for the choice (14, 22) and discussion of its limitations (14). The added value of radiomics should be evaluated not only statistically—for example, the significance of an AUC increase (33, 43) —but also in terms of cost-effectiveness (17), such as quality-adjusted life years (QALY) (21, 35, 42, 46, 98). Finally, the tool's performance should be assessed within the context of its intended clinical use (24).

### 3.3 Biological validation

Biological validation refers to establishing a link between imaging biomarkers and tumor biology (137). Although not mandatory for clinical application (97), it is highly beneficial to show that an intervention's effect on a surrogate endpoint (e.g., reduced GLCM contrast after radiotherapy, reflecting decreased heterogeneity) reliably predicts its effect on the true clinical endpoint (e.g., tumor control after radiotherapy). This process involves correlating radiomic with non-radiomic features (21, 33, 54), confirming biological plausibility (20), and linking macroscopic tumor phenotypes captured by radiomics to microscopic tumor biology (46, 60, 93). Biological validation reduces the risk that radiomic features are selected by chance or reflect only the development dataset (97).

Evidence has been found linking radiomic information to tumor biology, such as its association with EGFR mutation status (134). Sun et al. (238) validated a radiomic signature from contrast-enhanced CT for tumor-infiltrating CD8 cells, related to the tumor-immune phenotype, using a gene expression signature. Preclinical studies may explore more closely the association of radiomic features with specific cell morphology and molecular pathways (97, 239) using diverse modalities such as Cone Beam CT (240), PET and MRI (241), Photon counting CT (242). Gene expression changes were linked to radiomic signatures by analyzing tumor models in wild-type vs. knockdown mice and correlating the results with patient data (243). Despite promising findings, linking biology and radiomics remains difficult (244) because of genetic complexity, indirect links to phenotype, and numerous correlated parameters requiring multiple comparisons (2, 97).

### 3.4 Regulatory framework

In the European Union (EU), The European Medical Devices Regulation (EU) 2017/745 (EU MDR) and *in vitro* Diagnostic Medical Devices Regulation (EU) 2017/746 (EU IVDR) in combination with the General Data Protection Regulation (EU) 2016/679 (GDPR) contain requirements for AI (245). The EU proposed a regulation called “The Artificial Intelligence Act (AI Act)” (246) aiming at introducing a common regulatory and legal framework for AI. Healthcare AI applications, as per the AI Act, are high-risk systems in that they may pose significant threats to health.

#### 3.4.1 USA

The US Food and Drug Administration (FDA) 510(k) is a premarket submission to demonstrate safety of a device, where in-scope and out-scope applications of the product must be clearly highlighted as well as the possible behavior of the product in the so-called edge cases, i.e., cases lying on the limits of the applicability boundaries of that given product (123). The FDA together with Health Canada, and the United Kingdom's Medicines and Healthcare Products Regulatory Agency (MHRA) issued the Good Machine Learning Practice (GMLP) for medical device development: guiding principles” where they jointly identified 10 guiding principles (247).

#### 3.4.2 Japan

The Japanese Pharmaceuticals and Medical Devices Agency 's Scientific Committee published “Issues and recommendations on AI-based medical diagnosis systems and medical devices” (248). Then the Japanese cabinet approved the Regulatory Reform Implementation Plan in June 2021, which summarized various policies to address most of the issues to be tackled in the future.

#### 3.4.3 China

In July 2021 the National Medical Products Administration (NMPA, formerly the China Food and Drug Administration, or CFDA) issued the “Guideline on Artificial Intelligence Medical Devices” (249), a guideline paying attention to the AI medical device life-cycle process. Unapproved or low-maturity AI-based medical software is classified as class II or III, dipending on whether it involves assisted decision-making. High-maturity AI-based medical software devices must follow the Medical Device Classification Catalog.

## 4 Future directions

Several barriers slow the translation of radiomics into practice. These include the absence of standardized imaging acquisition and preprocessing protocols (244), the frequent use of small or single-center datasets that limit generalizability (250), and the broader need for large, diverse, and well-annotated cohorts to enable robust model development and validation. Additional challenges involve demonstrating clinical utility in real-world settings and integrating radiomics outputs seamlessly into existing workflows (244).

A key obstacle remains the limited availability of large annotated datasets—restricted by privacy concerns, proprietary limitations, and heterogeneous data formats. This highlights the importance of multi-institutional collaboration and the creation of centralized imaging repositories to ensure access to diverse, high-quality data (251).

Challenges arising during model development can also be mitigated through methodological best practices. Data imbalance may be addressed via oversampling algorithms, while dimensionality reduction and regularization can help prevent overfitting. It is essential to explicitly report measures taken to prevent information leakage. Data leakage is the situation in which information available during evaluation is unintentionally included in the training process, causing the training and evaluation data to no longer be truly independent (252). Because data leakage occurs during training, it can artificially boost the model's reported performance, making the evaluation metrics misleading compared to how the model will truly perform during inference Leakage controls include such as defining an independent test set *before* any normalization, feature selection, hyperparameter tuning, or model training (16, 18, 19, 43). The test set should be used only once for the final model evaluation to avoid biased or overly optimistic results (14).

Model interpretability, a major prerequisite for clinical adoption, can be strengthened through methods such as activation maps, feature-importance analyses (14, 22), Shapley additive explanations (102, 120), and systematic failure analysis of misclassified cases (47). Transparency and reproducibility can be further enhanced by sharing code, raw data, and complete methodological descriptions (251). As discussed in Sections 1.3 and 1.5, the availability of open-source libraries has already played an important role in facilitating radiomics research.

External testing by independent teams in radiomics helps verify that a model's performance is not limited to one dataset or experimental setup, demonstrating its potential for wider clinical use. However, such testing is still conducted by only a small number of research groups (253)

Another major challenge for clinical implementation is establishing biological plausibility. This involves demonstrating robust and reproducible relationships between radiomic phenotypes and the underlying microscopic biology (33, 42, 43, 46, 60, 93), While preclinical studies can investigate these links more directly, clinical studies may assess associations between radiomic features and histopathology, gene mutations or expression profiles, and metabolic characteristics (254).

Guidelines in radiomics offer structured pathways to overcome these barriers. However, their adoption remains limited. A meta-research analysis of 117 radiomics studies found that only 7 (6%) reported adherence to at least one quality or reporting framework (14). Similarly, another study reported that only 2 of 33 papers (6%) included a self-reported quality score (73). Comparable findings have been echoed by other authors (122, 255).

Based on the findings of our systematic review, we can synthesize the high consensus recommendations divided into 5 major areas: (a) Study Design: Carefully define the study rationale, objectives, and outcomes, ensuring the dataset is of adequate size and quality, (b) Data Workflow: Use standardized protocols for image acquisition, reconstruction, preprocessing, and feature extraction—following IBSI guidelines where applicable, (c) Model Development and Validation: Follow best practices for model development, including prevention of data leakage, dimensionality reduction, strategies to enhance model interpretability, and establish biological plausibility, (d) Transparency and Reproducibility: Publish results with sufficient methodological details to ensure rigor and generalizability and promote open science by sharing codes and data, (e) Quality and Compliance: Evaluate study compliance with relevant guidelines and regulations using appropriate quality metrics.

There remain several areas where further standardization through guidelines and recommendations is needed. First, this review focuses exclusively on handcrafted features, even though deep learning–based features are receiving increasing attention for their ability to capture more abstract and high-level image representations (256). Second, in studies involving rare diseases, datasets are often small and highly imbalanced. Existing guidelines should therefore be expanded to address strategies for managing class imbalance and to provide recommendations on appropriate algorithms, model architectures, and parameter settings for such scenarios. Furthermore, federated learning has recently emerged as a promising approach to reduce the reliance on large, centrally collected datasets (257). However, this technique also requires the development of dedicated standards and guidelines to ensure consistency, reproducibility, and data security across institutions.

## 5 Conclusions

Radiomics holds the promise of delivering clinically meaningful imaging biomarkers and represents an important step toward truly personalized medicine. To achieve this goal, researchers must first clearly define the study objective—particularly the specific type of imaging biomarker under investigation—and then follow a rigorously standardized workflow aligned with established guidelines. In this systematic review, we identified five major guidelines and regulatory frameworks developed in recent years for radiomics and AI, which collectively provide the research community with essential direction for designing, conducting, and validating radiomic studies capable of producing biomarkers suitable for clinical implementation.
